# Outcomes and Fate of the Distal Landing Zone Compared Between Prosthetic and Autologous Grafts After Infra-Inguinal Graft Occlusions

**DOI:** 10.3389/fsurg.2022.811126

**Published:** 2022-02-22

**Authors:** Ulrich Rother, Marc Gruber, Christian-Alexander Behrendt, Josefine Günther, Werner Lang, Alexander Meyer

**Affiliations:** ^1^Department of Vascular Surgery, Friedrich Alexander University Erlangen-Nuremberg, Erlangen, Germany; ^2^Department of Vascular Medicine, Research Group GermanVasc, University Heart and Vascular Centre UKE Hamburg, University Medical Centre Hamburg-Eppendorf, Hamburg, Germany

**Keywords:** infrainguinal bypass, graft failure, prosthetic graft, vein bypass, limb-threatening ischemia

## Abstract

**Background:**

Due to an increasing life expectancy, more and more patients experience the failure of peripheral arterial revascularization. This study aims to investigate patients treated for the failure of infra-inguinal bypass grafts, and to investigate the interaction of different bypass materials [great saphenous vein (GSV) and polytetrafluoroethylene (PTFE)] and the further outcome.

**Methods:**

Retrospective single-center analysis of consecutive patients treated for acute or chronic occlusion of infra-inguinal bypasses at a university hospital was conducted. Hospitalizations from 1st January 2010 through 31st December 2019 were included. Perioperative parameters from the index operation including graft material (prosthetic vs. autologous) were assessed. After bypass occlusion, the grade of ischemia, as well as the distal landing zone of the redo bypass compared with the primary bypass was investigated.

**Results:**

In this study, 158 (65% men and 35% women with a m mean age of 70.5 years) eligible patients were included (57% vein and 42% prosthetic bypass grafts). After graft occlusion, 47% of the patients presented with symptoms of acute limb-threatening ischemia, 53% with symptoms of chronic leg ischemia. The rate of acute limb-threatening ischemia was significantly higher when prosthetic graft material was used during the index operation (*p* =0.016). Additionally, in case of reoperation, the landing zone of the redo bypass was significantly more distally located after occlusion of prosthetic bypass graft (*p* = 0.014)

**Conclusion:**

Occlusions of prosthetic bypass grafts were associated with significantly higher rates of acute symptoms compared with vein grafts. Additionally, a shift to a more distal landing zone was recognized after the failure of a prosthetic bypass graft during the redo bypass operation.

## Introduction

More than 200 million people worldwide are known to be affected by peripheral artery disease (PAD) (1). This is the result of increasing numbers of patients with diabetes mellitus on the one hand, however on the other hand due to demographic changes. Accordingly, the number of patients treated either by endovascular procedures or open surgical procedures is increasing (2).

Depending on their clinical presentation, the patients with graft occlusion can either be managed conservatively, revascularized by an endovascular or open surgical approach, or undergo a primary major amputation.

In cases where bypass operations are necessary for revascularization, the great saphenous vein (GSV) is recommended as graft material; prosthetic bypass grafts (such as polytetrafluoroethylene (PTFE)/expanded PTFE (ePTFE) grafts) should only be considered if there is no vein material available, and the revascularization cannot be done by an endovascular approach (3).

Although the treatment options, as well as the durability of the interventions, are evolving, more and more patients experience the failure of revascularization procedures, due to increased expectancy of life (4). Therefore, it becomes necessary to consider the consequences of graft occlusion, which in turn should influence decision-making in revascularization procedures, especially in patients with intermittent claudication (IC).

This study aims to investigate patients treated for the failure of infra-inguinal bypass grafts, and to investigate the interaction of different bypass materials (GSV and PTFE) and the further outcome.

## Materials and Methods

### Patients

A retrospective single-center analysis of consecutive patients treated for acute or chronic occlusion of the infra-inguinal bypass at a university hospital was performed. All patients between 1st January 2010 and 31st December 2019 were screened for meeting the inclusion criteria. The study was conducted in congruence with the declaration of Helsinki, adhered to the STROBE guidelines, and was further approved by the local ethics committee (number 40_21 Bc) (5). Written informed consent was not deemed necessary due to the study's retrospective character.

### Inclusion Criteria

All patients treated for chronic (Fontaine stages II-IV) or acute (Rutherford stages I-III) due to the first occlusion of an infra-inguinal bypass graft were included in this study. Only GSV (single-segment, all implanted in reversed technique) or ePTFE bypass grafts used during the index operation were included. Excluded were other-cause acute or chronic ischemia, such as cardiac embolism, arterial thrombosis, occlusions due to failed interventional or endovascular revascularization of occluded grafts, as well as other occluded bypass grafts than GSV or ePTFE (e.g., small saphenous vein or ovine prosthesis).

### Study Design and Clinical Parameters

Different clinical parameters were assessed, besides the baseline values such as age in years, dichotomized sex, and comorbidities (current smoker, end-stage renal disease, arterial hypertension, history of diabetes mellitus longer than 2 years, and dyslipidemia). All data concerning the index bypass operation (first bypass operation) were retrospectively collected, including the time point of operation, the bypass graft material (the choice of the bypass material was in all cases a vein first approach if sufficient material was available), localization of the proximal and distal anastomosis as well as the anticoagulation regime after the bypass operation. At the time point of study inclusion (first bypass graft occlusion), the degree of ischemia was assessed. Dependent on clinical presentation, this was either a chronic situation, graded according to the Rutherford classification (Stages 3–6), or an acute occlusion, graded according to the Rutherford classification (I-III). The bypass occlusion was primarily detected by duplex ultrasound, however always verified by either computed tomography angiography (CTA) or digital subtraction angiography (DSA).

Further treatment was investigated such as redo bypass, lysis therapy, endovascular revascularization, or major amputation. In cases where redo bypasses were indicated, the level of the distal anastomosis was assessed. Therefore, only shifts from above-the-knee to below-the-knee or a tibial distal landing zone were counted as a shift of the level of anastomosis during further analysis. The follow-up of the bypass patients (vein and PTFE) was routinely done by ABI every 6 months, in cases of vein bypasses additional duplex ultrasound was performed. Routine therapy post bypass was done by single antiplatelet therapy, according to current literature in cases of small vein calibers (<4mm) or reduced outflow by anticoagulation (warfarin or direct oral anticoagulation, DOAC). However, in cases of reduced outflow and prosthetic bypasses, it is done by double antiplatelet therapy (6, 7). Reduced outflow was defined as occlusion of the pedal arch, as reported by Kawarada et al. (8).

### Statistical Analysis

All statistical calculations were performed using SPSS 21 (SPSS Inc., Chicago, IL, USA). The normality of the values was assessed by the Shapiro-Wilk test. In the case of normal distribution, the mean and *SD* were used, in cases of skewed distribution median values together with minima and maxima were used. For qualitative factors, absolute and relative frequencies are given. The comparison of two independent groups was performed using the chi-squared test (comparison of group differences in Table 1), Fisher's exact test (in cases of small sample sizes of group differences in Table 1), the Mann-Whitney *U* test (comparison of independent median values such as BMI in Table 1), or a two-sample *t*-test (comparison of age between groups in Table 1). To investigate the differences in patency and survival rates, Kaplan-Maier curves and the log-rank test were used to estimate the time to occlusion between venous and prosthetic bypass grafts. A Cox proportional hazard regression model was conducted to investigate the influence of different variables on the time to bypass occlusion (including the parameters anticoagulation, bypass material, and bypass anatomy). For all statistical tests, *p* < 0.05 was considered to show a statistically significant difference.

**Table 1:** Patient characteristics and comorbidities.

**Patients**	**Venous bypass**	**Prosthetic bypass**	***p*** **-value**
Age (years; mean, SD)	70.3 (11.3)	71.0 (9.8)	0.121
Gender (♀;♂); n (%)	60;30 (66.7;33.3)	41;25 (62.1;37.8)	0.557
Body mass index (mean, SD)	27.2 (4.5)	26.6 (3.9)	0.977
Smoker; n (%)	42 (46.7)	38 (57.6)	0.178
Renal end stage disease; n (%)	2 (2.2)	1 (1.5)	0.751
Diabetes mellitus; n (%)	39 (43.3)	22 (33.3)	0.206
Dyslipidaemia; n (%)	38 (42.2)	37 (56.1)	0.087
Hypertension; n (%)	79 (87.8)	57 (86.4)	0.794

## Results

### Patients and Procedure Characteristics of the Index Operation

A total of 158 (102 men and 56 women with a mean age of 70.5 (range 36–98 years) consecutive patients treated for bypass graft occlusion were included in this study. The patient's comorbidities are shown in Table 1. There were found no significant differences between the prosthetic and venous bypass groups concerning the comorbidities.

The indication for the index operation was intermittent claudication in 57 cases, rest pain in 16 patients, and chronic wounds in 75 cases. Furthermore, 10 patients were operated on due to acute ischemia. In 90 cases the graft material was the great saphenous vein, in 66 cases an ePTFE bypass graft, in two cases the graft material was retrospective not assessable. In those patients presenting with acute limb ischemia, seven were treated by venous bypass and three by prosthetic bypass. The anticoagulation regime as well as the perioperative characteristics after the index operation are displayed in Table 2.

**Table 2:** Periprocedural characteristics of the index operation.

**Periprocedural characteristics**	
Rutherford Stage; n (%):	
3	39 (25)
4	22 (14)
5/6	87 (55)
Acute limb ischemia	10 (6)
Bypass material; n (%):	
Great saphenous vein	90 (57)
Prosthetic (ePTFE)	66 (42)
Not assessable	2 (1)
Anticoagulation; n (%):	
ASS	68 (43)
Clopidogrel	11 (7)
Dual antiplatelet therapy	15 (10)
Warfarin	22 (14)
Direct oral anticoagulation	7 (4)
None	35 (22)
Bypass anatomy; n (%):	
Above-the-knee	54 (34)
Below-the-knee	53 (34)
Tibial	51 (32)
Proportion of below-the-knee PTFE grafts	40 (25)
Ankle brachial index (mean, SD)	
Preoperative	0.37 (0.23)
Postoperative	0.83 (0.15)

### Time-to-Occlusion After the Index Operation

**Figure 1:**
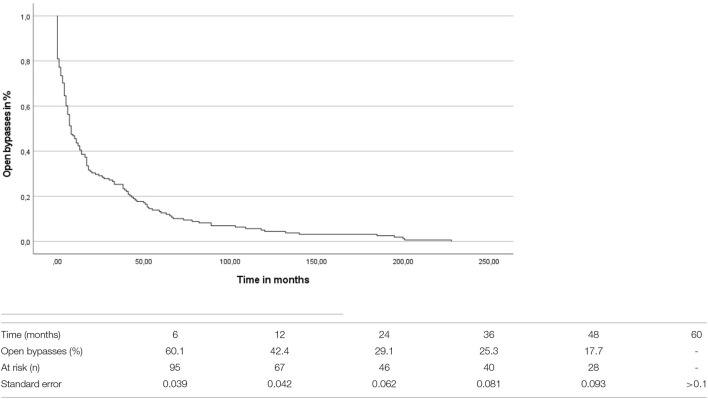
Kaplan-Meier curve of the time to bypass occlusion.

The overall mean time-to-occlusion was 26.55 (*SD* = 42.98) months (compare Figure 1). By comparing the period to occlusion between venous and prosthetic bypass grafts irrespective of the level of the distal anastomosis there was no significant difference found between the groups (log-rank test: *p* = 0.251). A Cox proportional hazard regression model was conducted concerning the time-to-occlusion including the anticoagulation regime, the bypass material as well as the different bypass localizations. Significant influence of the distal anastomosis level on graft patency was detected, with distal location as an independent predictor of occlusion [hazard ratio (HR) 1.283, CI 1.044–1.577, *p* = 0.018]. The bypass material [odds ratio (OR)0.97, CI.659–1.429, *p* =0.878] as well as the anticoagulation (OR 1.012, CI.871–1.176, *p* = 0.875) had no significant influence on the time to bypass occlusion in the Cox-regression analysis.

### Bypass Occlusion

The degree of ischemia at the time point of bypass occlusion was assessed. The presence of proximal/inflow stenosis could be ruled out by CT-Angiography, which was performed in each patient upon presentation. Symptoms of acute limb-threatening ischemia after bypass occlusion were shown by 74 patients. Chronic symptoms due to the bypass occlusion were presented by 84 patients (for detailed information compare Table 3). Further analysis showed a significantly higher proportion of patients presenting with acute ischemia after occlusion of prosthetic bypass graft (venous group: 38.9%, prosthetic group: 57.6%; *p* = 0.016). Similarly, the occlusion of tibial bypass grafts led to significantly more acute ischemic situations than other bypass anatomies (*p* = 0.046). However, even in this subgroup, the impact of the prosthetic material on the rate of acute ischemic situations was significant (*p* = 0.026).

**Table 3:** Periprocedural characteristics of the revision operation and clinical presentation at bypass occlusion.

**Overall time to bypass occlusion**	**26.55 (SD 42.98) months**
**Clinical presentation after bypass occlusion**	
Acute limb threatening ischemia; n (%)	74 (47)
Rutherford I	22 (14)
Rutherford II	36 (23)
Rutherford III	16 (10)
Chronic limb ischemia; n (%)	84 (53)
Rutherford stage 3	19 (12)
Rutherford stage 4	16 (10)
Rutherford stage 5/6	49 (31)
**Revision characteristics**	
Symptoms of acute limb threatening ischemia; n (%)	74 (47)
Type of therapy after graft occlusion n (%):	
Redo bypass Open surgical bypass thrombectomy	88 (56) 31 (20)
Catheter-directed thrombolysis (vein grafts only, combined with balloon angioplasty of demarking stenosis)	4 (3)
Major amputation	20 (13)
Conservative	15 (10)
Bypass anatomy; n (%):	
Above-the-knee	10 (11)
Below-the-knee	34 (39)
Tibial	44 (50)
Bypass material; n (%):	
Vein (contralateral Great saphenous or arm vein)	1 9 (22)
Prosthetic (ePTFE)	69 (78)

### Revision Operation

The revision procedures after bypass occlusion were additionally analyzed (compare Table 3). In 88 (55.7%) cases a redo bypass operation became necessary. In brief, 31 bypass grafts were revascularized by bypass thrombectomy, followed by a patchplasty of the anastomosis, in 20 cases primary major amputation was necessary, 4 were treated by catheter-directed thrombolysis and 15 patients were treated conservatively without revascularization. The choice of the bypass material during the index operation had no significant influence on the primary amputation rate in this cohort (primary amputation venous group 13.3%, prosthetic group 12.1%; *p* =0.823). In cases where redo bypass operations became necessary, the level of the distal anastomosis was evaluated and compared to the level of the anastomosis of the index operation. By comparing the levels of the distal anastomosis between the prosthetic and venous bypass group, the level of the anastomosis during the revision operation was significantly more distal after occlusion of prosthetic bypass graft (*p* = 0.014).

### The Outcome of the Revision Procedures

After successful revascularization the mean ankle brachial index improved significantly from 0.26 (*SD* = 0.25) to 0.82 (*SD* = 0.19) (*p* < 0.001). Among the initial included 158 patients, 27 died during the follow-up.

The subgroup analysis of the redo bypass operations showed mean patency of 17.8 (*SD* = 30.4) months during further follow-up. However, out of these 88 patients, 12 died during the follow-up. A separate analysis of the outcome of tibial reconstructions with respect to the used material during the revision operation showed no significant differences between prosthetic and venous material (mean patency venous bypass 15.6 (*SD* = 14.6), mean patency prosthetic bypass 15.1 (*SD* = 20.2), *p* = 0.952).

## Discussion

Several published studies aimed at the investigation of patency rates after open surgical bypass procedures (9–12). They mainly focused on the influence of different preexisting conditions in the patency or investigated the type of the procedure or material on the long-term patency. However, literature on the further proceedings after peripheral bypass occlusion is relatively scarce.

The study of Bodewes et al. for example investigated the perioperative outcomes of infra-inguinal bypass operations in patients with and without prior ipsilateral revascularization, either by open surgical or endovascular procedures (10). In their study, 21% of included patients underwent secondary bypass and 17% of the patients had previous endovascular interventions. According to their results, the previous revascularization was associated with worse bypass results in terms of infection and reintervention rates. Most of the previously published literature yields similar results, some show worse outcomes for the secondary bypass after failed endovascular revascularization, however, some cannot see the influence of the prior revascularization (13–16). As this is well documented in recent literature, outcomes of bypass surgery following endovascular revascularization were outside the topics of the present study, and therefore these patients were excluded from the analysis.

Accordingly, in this study, we specifically focused on the impact of graft failure on the potential redo-procedures in terms of the anatomic level of the distal anastomosis in the case of secondary bypass. Furthermore, we addressed the influence of the initially used graft material on the grade of ischemia of the limb at patients in hospital presentation. To exclude a potential selection bias, only the first occlusion of the particularly first bypass implanted on the index leg was included for analysis. The decision-making with regard to the graft material was left to the operating surgeon's discretion, whereas an “autologous first” policy is followed at our institution. This means that in cases of prosthetic graft implantation, the GSV is intraoperatively explored as a rule, and ePTFE grafts are used only in GSV diameters <3 mm or presence of heavily varicose veins or sclerotic segments. As exclusively graft occlusion are evaluated in this review, the percentage of ePTFE grafts (42%) may be overrepresented compared to the whole collection of bypass procedures due to their known reduced patency rates. Regarding the redo surgery, contralateral GSVwas used as primary graft material for redo procedures, when available, the second choice of graft material was the usage of arm vein grafts, which are however only suitable in a minor percentage of patients in our experience. Against this background, in everyday practice, the majority of patients will require a synthetic graft as material for revision surgery, which was done, when indicated, in both acute and chronic ischemia.

Interesting is the further outcome of patients subsequent to graft occlusion in the present study. Although only half of the patients presented with acute limb-threatening ischemia, just a small minority (10%) could be managed conservatively without any revascularization procedures. This may be due to the fact that 75% of index procedures were performed for chronic limb-threatening ischemia, where the limb is dependent on graft perfusion. On the other hand, a low amputation rate (13%) following graft occlusion is comparable to the rate of patients manageable with conservative treatment, which implies, that the majority of limbs with graft occlusions can be salvaged by means of aggressive revascularization.

The present evaluation showed that patients receiving a prosthetic bypass graft during the first operation revealed–significantly higher rates of acute limb-threatening ischemia than patients with venous bypass grafts–in case of a bypass occlusion. One reason for this finding might be the more thrombogenic surface of the prosthetic bypass graft compared to the vein, which is known to lead more likely to the propagation of thrombus material into the native circulation; again, leading to an occlusion of the immediate outflow segment of the bypass (17). Similar findings were published by Jackson et al. (17). They found a significantly higher percentage of limb-threatening ischemia after bypass occlusion in patients treated with PTFE bypass compared to patients with venous bypass.

This aspect has been widely discussed in the literature, as some authors recommended as initial bypass material for suprageniculate bypass grafts routinely a prosthetic material to spare the great saphenous vein for secondary (e.g., infrageniculate) bypass reconstructions (12, 18–21). However, against this background, Klinkert et al. showed for example in their systematic review that the patency rate of the vein was superior at all study time intervals up to 5 years of investigation compared to ePTFE bypass grafts (11). Taking this into account, in addition to the above mentioned, even in the above-the-knee region a prosthetic bypass first approach would not be suitable. This aspect is additionally supported by our finding that there is a shift to a significantly more distal landing zone after occlusion of a prosthetic bypass graft compared to venous grafts during the redo bypass operation; this again supports the theory of thrombotic obstruction of the outflow after prosthetic bypass occlusion. These circumstances have already been reported before, however never been systematically investigated. By comparing the distal landing zones of the first bypass and the second bypass between prosthetic and vein grafts after occlusion, we could demonstrate that in cases of prosthetic graft occlusion, the distal landing zone significantly differs from vein grafts. Decision-making for the implantation of prosthetic grafts in the stage of claudication should therefore be critically evaluated for each patient. Our data clearly demonstrate the potential worsening of clinical state in case of graft occlusion, the need for more distal anastomosis and there is a low but present risk of amputation when the bypass fails.

There are some limitations of this study. Firstly, the study was conducted retrospectively including all-natural study limitations of this setting. However, to the best of our knowledge, the present study represents the largest in terms of sample size in graft occlusions. It is nonetheless important to consider that only patients with occluded bypasses were included, so prospective patency rates of autologous vs. prosthetic grafts could not be assessed, which was beyond the scope of this study.

## Conclusion

This study demonstrates that the occlusion of a prosthetic bypass graft was associated with a significantly higher rate of patients presenting with acute limb-threatening ischemia compared to vein grafts. Additionally, there was recognized a shift to a more distal landing zone after the failure of a prosthetic bypass graft during the redo bypass operation. This should be considered when indicating bypass operations with prosthetic graft material.

## Data Availability Statement

The original contributions presented in the study are included in the article/supplementary material, further inquiries can be directed to the corresponding author.

## Ethics Statement

The studies involving human participants were reviewed and approved by Ethics Committee of the University Erlangen-Nuremberg. Written informed consent for participation was not required for this study in accordance with the national legislation and the institutional requirements.

## Author Contributions

UR, WL, and AM: conceptualization. UR, C-AB, JG, and MG: methodology. UR and MG: formal analysis. MG, UR, JG, C-AB, and AM: investigation. UR, MG, C-AB, WL, and AM: writing—original draft preparation. UR and AM: writing—review and editing. All authors contributed to the article and approved the submitted version.

## Conflict of Interest

The authors declare that the research was conducted in the absence of any commercial or financial relationships that could be construed as a potential conflict of interest.

## Publisher's Note

All claims expressed in this article are solely those of the authors and do not necessarily represent those of their affiliated organizations, or those of the publisher, the editors and the reviewers. Any product that may be evaluated in this article, or claim that may be made by its manufacturer, is not guaranteed or endorsed by the publisher.
